# Localising OCTA changes induced by the isometric hand-grip test to the superficial retina in neovascular age-related macular degeneration

**DOI:** 10.1186/s40662-025-00459-9

**Published:** 2025-11-01

**Authors:** Matt Trinh, Yoh Ghen Tee, Judy Nam, Simon Chen, Gary Schiller, Jeff Friedrich, David Ng, Angelica Ly, Chris Hodge, Lisa Nivison-Smith

**Affiliations:** 1 School of Optometry and Vision Science, University of New South Wales, Sydney, NSW 2015 Australia; 2 Vision Eye Institute, Sydney, NSW Australia

**Keywords:** Isometric exercise, Optical coherence tomography angiography, Neovascular age-related macular degeneration, Retinal vascular flow, Choroidal vascular flow

## Abstract

**Purpose:**

This study uses optical coherence tomography angiography (OCTA) topographical cluster analysis to localise where vascular changes occur during the isometric hand-grip test (IHGT) in eyes with neovascular age-related macular degeneration (AMD).

**Methods:**

This prospective study included single eyes from 44 participants with neovascular AMD. Systemic blood pressure (BP) and macular 6 × 6 mm OCTA scans were obtained before the IHGT, during the IHGT, and after the IHGT. The main outcome was the change in processed OCTA signal (%), measured within high-density (126 × 126) grids and analysed by topographical clusters across the superficial retina, deep retina, and choriocapillaris. Results were compared against test–retest thresholds to differentiate true IHGT-induced changes from measurement variability.

**Results:**

The IHGT increased systolic (13.83 [3.28, 24.39] mmHg) and diastolic BP (7.04 [3.57, 10.52] mmHg; *P* < 0.01). Adjusted for test–retest thresholds, the IHGT increased processed OCTA signal (12.84 [8.49, 26.77] %, *P* < 0.0001) at nasal clusters in the superficial retina. These changes were moderately correlated with systolic BP increases (Spearman r = 0.43, *P* < 0.05), but not with diastolic BP. No changes were observed in the deep retina or choriocapillaris. Systemic BP and processed OCTA signal returned to baseline within 30 s after IHGT release.

**Conclusion:**

Hand-squeezing temporarily increases processed OCTA signal in the nasal superficial retina. This response may serve as a valuable marker of vascular function. Consequently, caution is warranted when interpreting OCTA following BP changes, such as those induced by physical activity or medication changes.

**Supplementary Information:**

The online version contains supplementary material available at 10.1186/s40662-025-00459-9.

## Background

Optical coherence tomography angiography (OCTA) provides a non-invasive and cost-efficient method for imaging neovascularisation in diabetic retinopathy, pachychoroid spectrum disease, age-related macular degeneration (AMD), and various other retinal diseases [1–3], supplementing the current gold-standard, intravenous angiography [4, 5]. However, the clinical adoption of OCTA lags behind other retinal imaging modalities [6]. In part, this is due to the dynamic nature of vascular flow, which can introduce significant variability in OCTA visualisation and make it challenging to distinguish true vascular changes from measurement noise. Augmenting visualisation of the retinal and choroidal vasculature could thus improve the clinical utility of OCTA and facilitate broader uptake in practice and research.

Recently, it was demonstrated in eyes with central serous chorioretinopathy [7–9] that OCTA visualisation of retinal and choroidal vessels can be augmented by exploiting the sympathomimetic effects of a simple, in-office isometric hand-grip test (IHGT) [10, 11]. To our knowledge, the specific retinal or choroidal regions affected by these changes remain unclear, and the effect has not yet been reproduced in other disease populations such as AMD, which also involves widespread retinal and choroidal vascular dysfunction [12]. Identifying the precise vascular regions influenced by the IHGT may provide further insights into the underlying physiological mechanisms involved. Furthermore, reproducing these findings in AMD—the leading cause of blindness in the developed world [13]—could support more widespread use of sympathomimetic augmentation to assess vascular function.

This pre-post ‘interventional’ study localises the effects of the IHGT (the ‘intervention’) on the retina and choroid, using OCTA topographical cluster analysis. The findings will provide insights into physiological, acute vascular stress responses and assess the potential utility of the IHGT for augmenting OCTA visualisation and assessing vascular function.

## Methods

This prospective, pre-post interventional study was a follow-up to a pilot crossover trial (ACTRN12621001522808), conducted in a separate population [14]. A single-group, non-crossover design was chosen, as the previous crossover trial demonstrated no period effect of the IHGT on OCTA signal [14].

Participants were recruited using convenience sampling of patients undergoing intravitreal anti-vascular endothelial growth factor treatment for any neovascular eye disease at two private ophthalmology centres—Vision Eye Institute at Chatswood and Hurstville, Sydney, Australia. Recruitment started on the 29th March 2023 and concluded on the 15th February 2024 upon meeting the pre-determined target sample size of 39 single eyes, calculated based on previous work [9].

Participants were eligible for inclusion if they had neovascularisation in either eye due to AMD. Neovascularisation was initially diagnosed using dye-based angiography and OCT. If both eyes met the inclusion criteria, then one eye was randomly selected for analysis using simple randomisation. Those with other retinal-choroidal diseases, such as diabetic retinopathy or pathological myopia, were excluded from analysis [15]. Scans were also excluded if they had significant imaging artefacts, e.g., from eye movement or blinking [16], or a signal strength index less than 6. No minimum grip strength was required for inclusion. All participants provided written informed consent for their deidentified data in research, which was further approved by the Biomedical Human Research Ethics Advisory Panel of the University of New South Wales (HC220761) and conforming to the tenets of the Declaration of Helsinki.

### Testing equipment

Systemic blood pressure (BP; systolic, diastolic, and pulse rate) was measured using the Welch Allyn Connex 3400 ProBP sphygmomanometer (Welch Allyn, USA). Based on the practical time delay between each period of the testing protocol, successive measures of systemic BP were valid [17–19].

OCTA scans were acquired using the Cirrus HD-OCT 6000 (Carl Zeiss Meditec, Germany) macular 6 × 6 mm protocol, with eye-tracking and motion correction turned on.

The IHGT was performed using the Jamar Smart Digital Hand Dynamometer (HMG Direct, Australia). As right-hand IHGT use and greater maximum absolute grip strength were independently associated with greater increases in systemic BP [14], participants were instructed to perform the test using their right-hand at maximum grip strength. The dynamometer was held with the elbow flexed at 90° and resting on the right lap [20, 21]. A minimum of 30 s for holding and releasing the IHGT was selected for practical reasons, to minimise time-disruption to clinical workflow and because previous physiological studies showed that the sympathomimetic pressor response can occur within approximately 30–60 s [10].

### Testing workflow

**Fig. 1:**
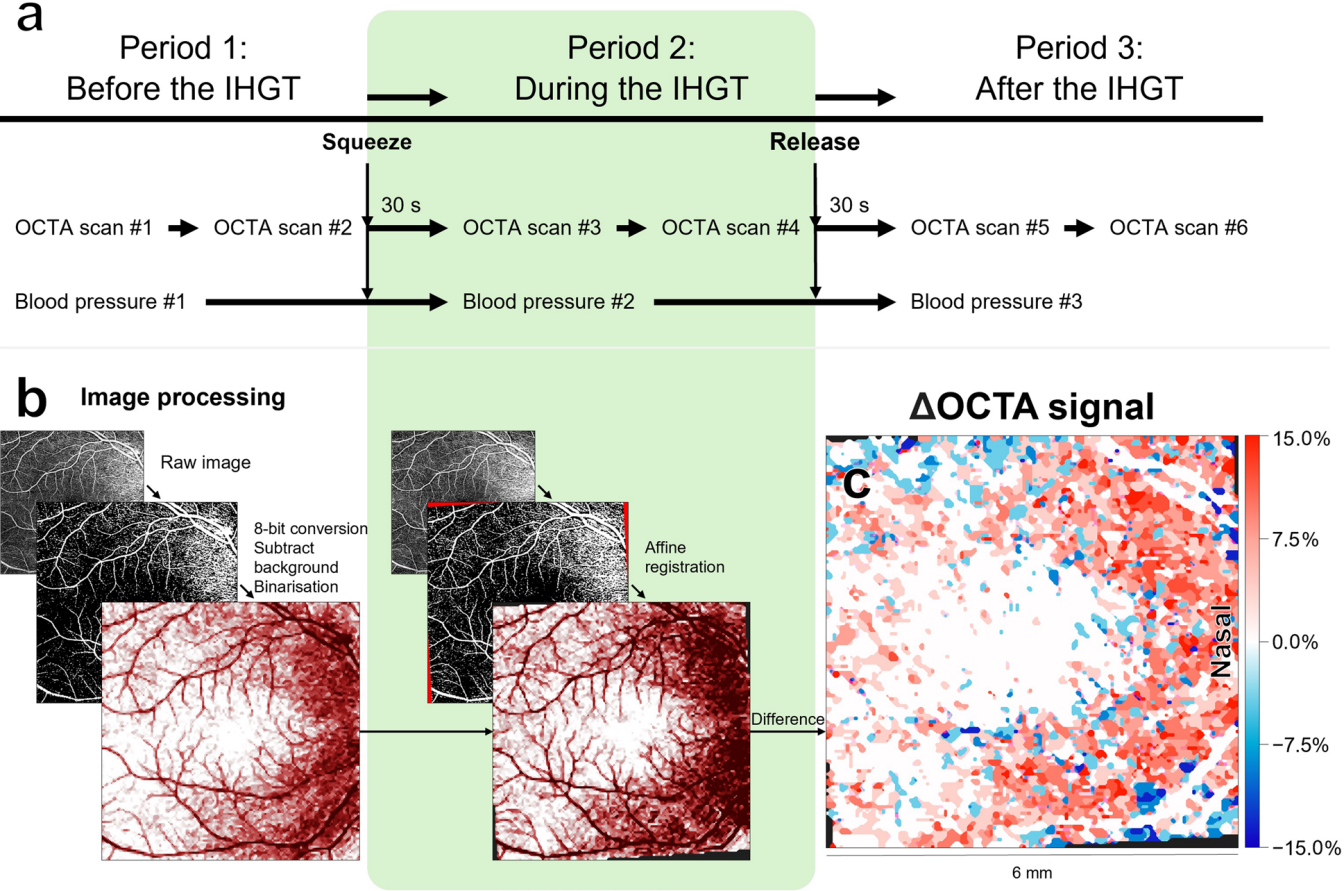
Summary of testing protocol and image processing. The testing protocol occurred across three periods: (**a**) before, during, and after the isometric hand-grip test (IHGT). In each period, two tracked optical coherence tomography angiography (OCTA) scans and one measure of systemic blood pressure (BP) were performed. Shaded in green is the period when the IHGT is held. Image processing and analysis then followed multiple steps (**b**): multiplication of the choriocapillaris OCTA by an inverted choriocapillaris OCT image, 8-bit conversion, background subtraction, binarization, affine registration, and masking of the superficial retina onto the deep retina and choriocapillaris. The main outcome measure was the change in processed OCTA signal (%). Note that after binarization, a colour scale was applied for improved visualisation; this step does not otherwise modify any underlying quantitative values. Changes (differences between periods) were then visualised as a topographical cluster map (**c**): red = increased processed OCTA signal, blue = decreased processed OCTA signal, and white = no change (e.g., particularly within larger retinal vessels). A maximum 1 × grid-size recursive median filter was applied to further minimise visual noise, without changing any quantitative values

All testing was conducted by a single trained operator per participant, immediately prior to their routine intravitreal anti-vascular endothelial growth factor treatment. All participants were pharmacologically dilated prior to testing. This study included three testing periods: before the IHGT, during the IHGT (held at maximum grip strength for at least 30 s), and after IHGT release (for at least 30 s; Fig. 1a).

1. In the first period before the IHGT, two OCTA scans and one measure of systemic BP were acquired.
2. In the second period during the IHGT, participants squeezed and held the IHGT for at least 30 s while two more OCTA scans and one measure of systemic BP were acquired.
3. In the third period after the IHGT, participants released the IHGT and after 30 s, two more OCTA scans and one measure of systemic BP were acquired.

### Image processing and analysis

Image processing and analysis then followed. Macular 6 × 6 mm OCTA en face scans of the superficial retina, deep retina, and choriocapillaris slabs were checked by authors MT and YGT, manually corrected where required, and excluded if there were significant errors present. The superficial retina was defined between the inner limiting membrane and outer border of the inner plexiform layer. The deep retina was defined between the outer border of the inner plexiform layer and outer border of the outer plexiform layer. The choriocapillaris was defined 24 to 49 µm below the retinal pigment epithelium, according to the Zeiss Cirrus Angioplex algorithm. The en face scan from each period with the higher signal strength (≥ 6/10) and overall image quality was extracted for further analysis.

OCTA image processing [22, 23] were performed as previous, transforming the raw OCTA image into processed OCTA signals (Fig. 1). Each choriocapillaris OCTA image was multiplied by its corresponding inverted greyscale OCT image to adjust for overlying signal loss from drusen. OCTA images across the superficial retina, deep retina, and adjusted choriocapillaris, then underwent 8-bit conversion, background subtraction with a 50-pixel rolling ball [24], and default auto-threshold binarization applied using ImageJ 1.53a (National Institutes of Health, Bethesda, MD, USA). Note that after binarization, a colour scale was applied for improved visualisation; this step does not modify any underlying quantitative values. Within-participant OCTA images were then registered using the affine transformation matrix based on manually-matched pixel coordinates (Python 3.12.6; Python Software Foundation, USA) [25]. Each deep retina and choriocapillaris OCTA image was overlaid with a mask of the superficial retina to adjust for shadowing and projection [26]. If the same pixels were missing in a majority of participants, due to image registration or masking, then these pixels were excluded from further analysis.

Then, OCTA topographical cluster analysis was performed as previously described (Fig. 1c) [27–29]. OCTA images were divided into 126 × 126 grids to generate topographical maps of change in processed OCTA signal (%) [27, 28], defined as the percentage of pixels with OCTA signal relative to the total area [30]. Change was defined as the difference in each grid’s OCTA signal between periods.

To localise and quantify the results while minimising noise, these changes were clustered into statistically similar groups using an adapted clustering method [29]. Specifically, K-Medians clustering was applied, with outlier clusters (i.e., those with fewer than 10 grids) removed and mode replacement used for adjacent values. Reiterations of clustering were performed using the Bayesian Information Criterion (BIC) to determine the optimal simple cluster model [31]. Starting with a single cluster, the number of clusters was increased only if BIC improvement showed strong (0.1) evidence per grid of a better fit, maintaining simpler models [32]. Clusters were separable by at least their 95th percentile values, with the cluster distance defined using these values. As a result, all clusters, except for one potential cluster crossing zero, were statistically significant (*P* < 0.0001).

Cluster maps were plotted with a colour scale, where red = increased OCTA signal, blue = decreased OCTA signal, and white = no change. A maximum 1 × grid-size recursive median filter was applied to further minimise visual noise, without changing any quantitative values. Clusters were also plotted graphically as histograms with axes denoting the cluster size (%; coverage of the macular scan area) and change in OCTA signal (%).

Analyses were repeated after removing values within test–retest thresholds, i.e., the median of differences between OCTA scans #1 and #2 in period 1 (before the IHGT). This was used to distinguish true IHGT-induced changes from measurement variability.

### Outcomes

The main outcome was the change in processed OCTA signal (%), compared before and during the IHGT, to determine the effect of the IHGT on OCTA signal. The secondary outcome was the (lack of) change in processed OCTA signal (%), compared before and after the IHGT, to establish adequacy of a 30-s IHGT wash-out (release) time.

### Statistical analyses

Default statistical significance was set as two-sided *P* < 0.05. Continuous variables were reported as mean [95% CI] or median [95% CI] as appropriate. Statistical analyses were performed using GraphPad Prism 10.0.3 (GraphPad, USA), Microsoft Excel v.2203 (Microsoft, USA), and Python 3.12.6 (Python Software Foundation, USA). All statistical comparisons assumed related data. Image quality was compared using the paired *t*-test. Systemic BP was compared using Tukey’s multiple comparisons. Cluster changes in OCTA signal were compared to zero using the one sample Wilcoxon test. Correlations were performed using non-parametric Spearman’s r and described as weak (0.1–0.39) or moderate (0.4–0.69) [33].

## Results

### Study population

Single eyes from 44 participants receiving treatment for AMD were included. Participants were 79.96 [77.35, 82.58] years of age, 70.45% female, 82.5% had cardiovascular-related disease, 93% right hand dominant, and maximum grip strength was 16.08 [13.66, 18.5] kg. Further descriptors are provided in Table 1.

**Table 1:** Baseline participant demographics and clinical characteristics

Demographics and characteristics	Values (44 eyes)
Age (years)	79.96 [77.35, 82.58]
Sex (% female)	70.45 (31/44)
Axial length (mm)	23.3 [22.88, 23.72]
Presence of cardiovascular-related disease (%)	82.5 (36/44)
Pseudophakia (%)	68.18 (31/44)
Caffeine intake in last 24 h (standard cups)	1.49 [1.12, 1.86]
Alcohol intake in last 24 h (standard drinks)	0.45 [0.21, 0.7]
Smoking (%, ever versus never)	38.64; n = 17/44
Time since waking (hours)	7.15 [6.42, 7.87]
Study eye (% right)	47.73 (21/44)
Dominant hand (% right)	93.02 (41/44)
Maximum grip strength (kg)	16.08 [13.66, 18.5]
Neovascularisation subtype (%)	Type 1, 31.82%; n = 14/44 Type 2, 47.73%; n = 21/44 Mixed or indeterminate, 20.45% (9/44)

From 44 study eyes, 14 (31.82%) had type 1 (subretinal pigment epithelium) neovascularisation, 21 (47.73%) had type 2 (subretinal) neovascularisation, and 9 (20.45%) had mixed or indeterminate neovascularisation. There were no statistically significant differences in demographic or clinical characteristics, systemic BP, or processed OCTA signal between neovascular subtypes (all *P* > 0.19). Image signal quality did not significantly differ between or within testing periods (*P* = 0.44).

### Effect of the IHGT on systemic BP

Firstly, to confirm the systemic effect of the IHGT, systemic BP was compared between period 1 (before the IHGT) versus period 2 (during the IHGT). There was a significant increase in systolic BP (13.83 [3.28, 24.39], *P* < 0.01) and diastolic BP (7.04 [3.57, 10.52], *P* < 0.0001; Table 2), confirming the systemic response.

**Table 2:** Comparison of systemic blood pressure (BP) before, during, and after the isometric hand-grip test (IHGT)

	Systemic BP			Tukey’s multiple comparisons *P* value	
Measure	Period 1: Before the IHGT	Period 2: During the IHGT	Period 3: After the IHGT	Period 1 vs. 2	Period 1 vs. 3
Systolic BP (mmHg)	**139.2 [130.7, 147.7]**	**153.1 [143.2, 162.9]**	143 [136.3, 149.6]	** < 0.01**	0.66
Diastolic BP (mmHg)	**80.32 [77.91, 82.73]**	**87.36 [83.59, 91.13]**	82.03 [79.28, 84.88]	** < 0.0001**	0.46
Pulse rate	73.91 [69.63, 78.19]	77.08 [71.82, 82.33]	75.08 [70.32, 79.74]	0.28	0.85

The IHGT induced significant increases in systolic BP (*P* < 0.05) and diastolic BP (*P* < 0.0001) between period 1 (before the IHGT) versus period 2 (during the IHGT). There was no significant difference between period 1 (before the IHGT) and period 3 (after the IHGT), demonstrating that 30 s after IHGT release was sufficient for BP to return to baseline. Bold font indicates statistically significant results

Comparison of periods 1 (before the IHGT) and 3 (after the IHGT) revealed no significant difference in systemic BP, demonstrating that 30 s after IHGT release was sufficient for BP to return to baseline.

### Effect of the IHGT on processed OCTA signal

**Fig. 2:**
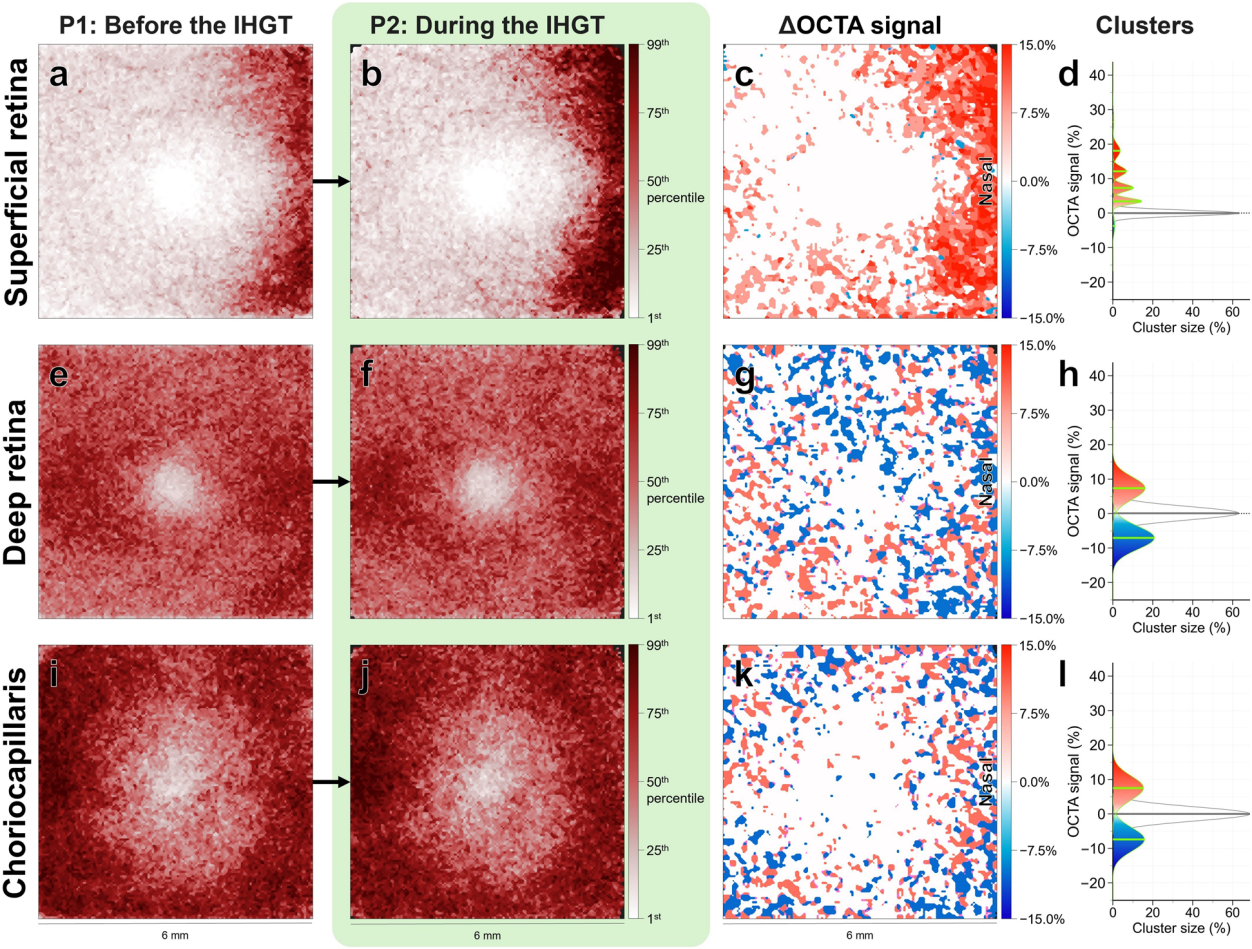
Processed optical coherence tomography angiography (OCTA) signal changes (%) by clusters. Processed OCTA signal changes (%) are shown for the superficial retina, deep retina, and choriocapillaris at (**a**, **e**, **i**) baseline period 1 (P1) before the isometric hand-grip test (IHGT), and (**b**, **f**, **j**) period 2 (P2) during the IHGT. Darker maroon grids = higher processed OCTA signal (%). The change in processed OCTA signal (**c**, **g**, **k**), i.e., the difference between P2 and P1, is shown in topographical cluster maps. Darker red grids = greater increases, darker blue grids = greater decreases, white grids = no change. All values denoted as median [95% CI]. Clusters were also plotted as histograms (**d**, **h**, **l**) to denote cluster size (%; coverage of the macular scan area) and change in processed OCTA signal (%). All clusters were statistically significant (*P* < 0.0001; maps, coloured areas; histograms, chartreuse lines) with exception of a single potential non-significant cluster crossing zero (maps, white areas; histograms, grey lines). Note the significant increase in processed OCTA signal at the nasal superficial retina, but otherwise likely noise in processed OCTA signal in the deep retina and choriocapillaris. Raw OCTA images in Figure S1

Secondly, to assess the effect of the IHGT on processed OCTA signal, topographical cluster analysis was performed to compare the first scans from periods 1 (before the IHGT) and 2 (during the IHGT). Qualitative assessment revealed a pattern of increased processed OCTA signal at the nasal superficial retina (Fig. 2a, b). Quantitative cluster analysis confirmed this, with multiple clusters showing a significant increase in processed OCTA signal predominantly in the nasal region (Fig. 2c), with magnitudes between 3.43 [1.92, 5.39] % to 26.42 [23.6, 36.24] % covering a total 35.7% of the macular scan (Fig. 2d).

No qualitative patterns or quantitative changes were observed in the deep retina (Fig. 2e–h) or choriocapillaris (Fig. 2i–l). There were some fluctuations in processed OCTA signal likely attributable to measurement variability, as indicated by the presence of symmetrically distributed clusters of increased and decreased signal with similar magnitudes, centred around zero (Fig. 2h, l).

### Effect of the IHGT on processed OCTA signal, adjusted for measurement variability

These results were further bolstered by repeated analyses comparing periods 1 (before the IHGT) and 3 (after the IHGT), excluding values within test–retest thresholds, i.e., the median of differences between the first and second OCTA scan in period 1 (before the IHGT), to distinguish true IHGT-induced changes from measurement variability.

**Fig. 3:**
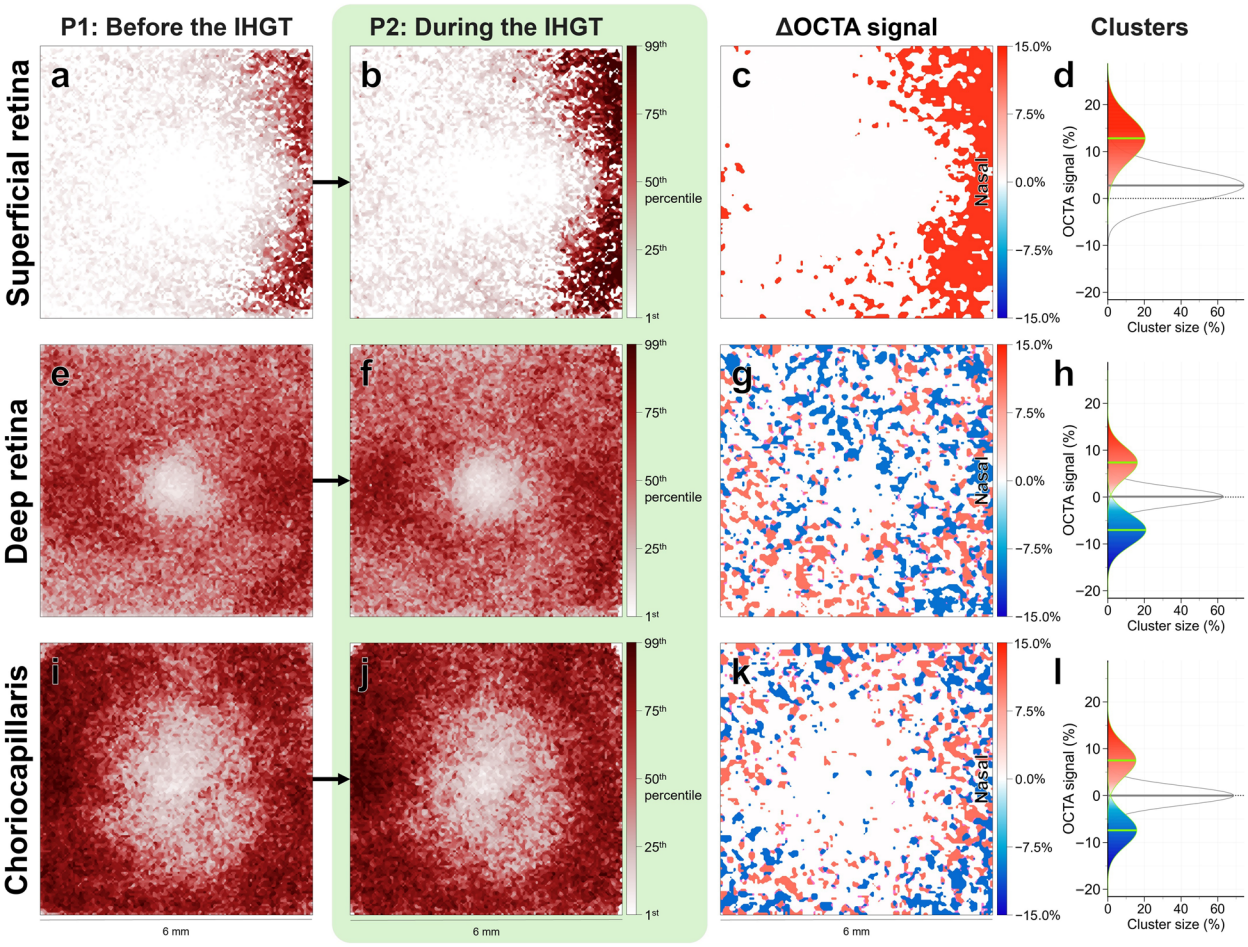
Processed optical coherence tomography angiography (OCTA) signal changes (%) by clusters, removing values within test–retest thresholds. Presentation as in Fig. 2, with additional removal of values within test–retest thresholds. Processed OCTA signal changes (%) are shown for the superficial retina, deep retina, and choriocapillaris at (**a, e, i**) baseline period 1 (P1) before the isometric hand-grip test (IHGT), and (**b, f, j**) period 2 (P2) during the IHGT. Darker maroon grids = higher processed OCTA signal (percentiles). The change in processed OCTA signal (**c**, **g**, **k**), i.e., the difference between P2 and P1, is shown in topographical cluster maps. Darker red grids = greater increases, darker blue grids = greater decreases, white grids = no change. All values denoted as median [95% CI]. Clusters were also plotted as histograms (**d, h, l**) to denote cluster size (%; coverage of the macular scan area) and change in processed OCTA signal (%). All clusters were statistically significant (*P* < 0.0001; maps, coloured areas; histograms, chartreuse lines) with exception of a single potential non-significant cluster crossing zero (maps, white areas; histograms, grey lines). Note the significant increase in processed OCTA signal at the nasal superficial retina, but otherwise likely noise in processed OCTA signal in the deep retina and choriocapillaris. Raw OCTA images in Figure S1

There was a similar pattern of increased processed OCTA signal at the nasal superficial retina (Fig. 3a, b), confirmed by quantitative cluster analysis as a uniform increase of 12.84 [8.49, 26.77] % covering 20.4% of the macular scan (Fig. 3c, d).

Consistent with the initial analysis, the deep retina (Fig. 3e–h*)* and choriocapillaris (Fig. 3i–l) did not exhibit distinctive patterns nor quantities of change beyond measurement variability. Symmetrically distributed clusters of increased and decreased processed OCTA signal with similar magnitudes, centred around zero, again were likely attributable to inherent measurement variability rather than true change (Fig. 3h, l).

### Relationship between BP and OCTA signal change

**Fig. 4:**
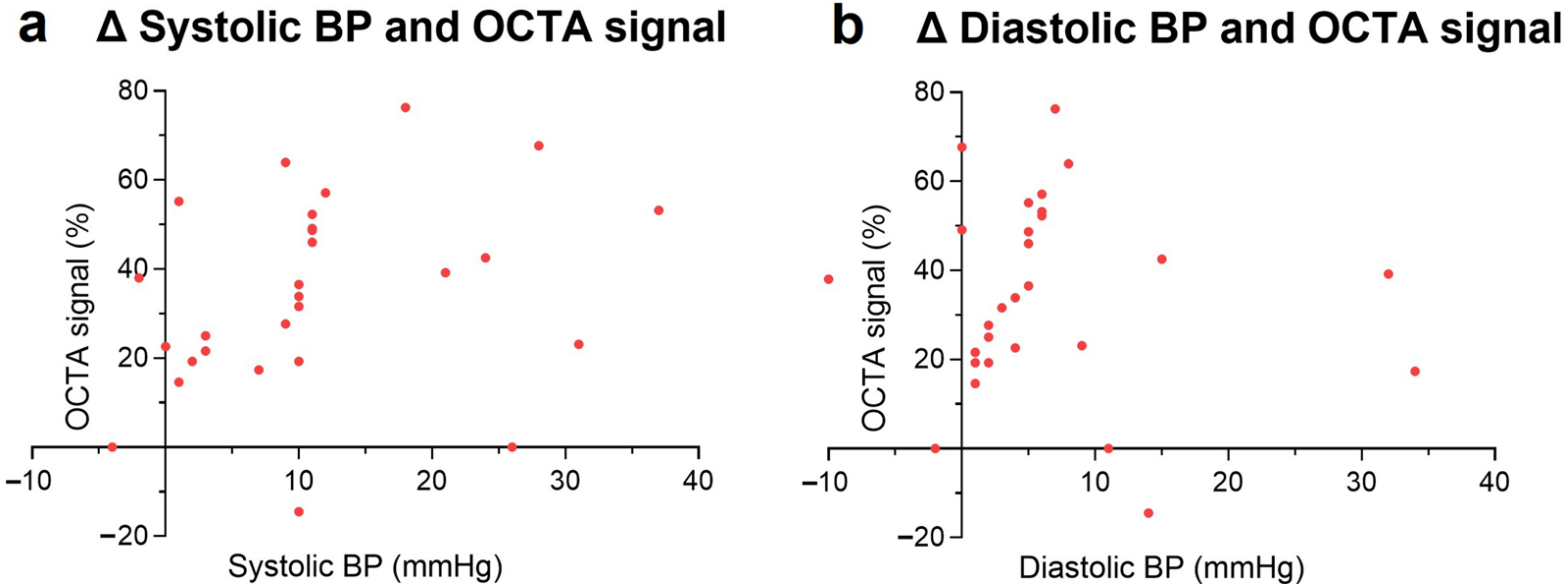
Change in systolic and diastolic blood pressure (BP) versus optical coherence tomography angiography (OCTA) signal. Changes in systolic BP and OCTA signal in the superficial retina (**a**), where a moderate, statistically significant correlation (Spearman’s r = 0.43, *P* < 0.05) is observed. Changes in diastolic BP and OCTA signal in the superficial retina (**b**) demonstrates a weak, non-significant correlation. Note that these represent singular sphygmomanometer measures, and other unmeasured factors may influence the OCTA signal changes observed

Given that only the superficial retinal slab showed significant OCTA signal change during the IHGT, we examined correlations between OCTA signal change and systemic BP parameters in this slab. The change in systolic BP was moderately and significantly correlated with the increase in processed OCTA signal (adjusted for measurement variability) in the superficial retina (Spearman’s r = 0.43 [0.06, 0.70], *P* < 0.05; Fig. 4a). In contrast, the change in diastolic BP showed a weak, non-significant correlation with OCTA signal change (Spearman’s r = 0.11 [–0.29, 0.47], *P* = 0.59; Fig. 4b). Note that these represent singular sphygmomanometer measures, and other unmeasured factors may also influence the OCTA signal changes observed.

### Effect of a 30-s IHGT wash-out on processed OCTA signal

To confirm that a 30-s IHGT wash-out (release) time was sufficient for processed OCTA signal to return to baseline, topographical cluster analysis was performed to compare the first scans from periods 1 (before the IHGT) and 3 (after the IHGT). While clusters indicated some differences between periods 1 and 3, the presence of symmetrically distributed clusters of increased and decreased processed OCTA signal with similar magnitudes, centred around zero, again suggested measurement noise rather than a true difference (Figure S2). Thus, 30 s after IHGT was sufficient for processed OCTA signal to return to baseline.

### One example of IHGT-induced change on processed OCTA signal

To provide a visual representation of IHGT-induced change, the following example of a 72-year-old female with systemic cardiovascular disease and a maximum grip strength of 11 kg is shown.

**Fig. 5:**
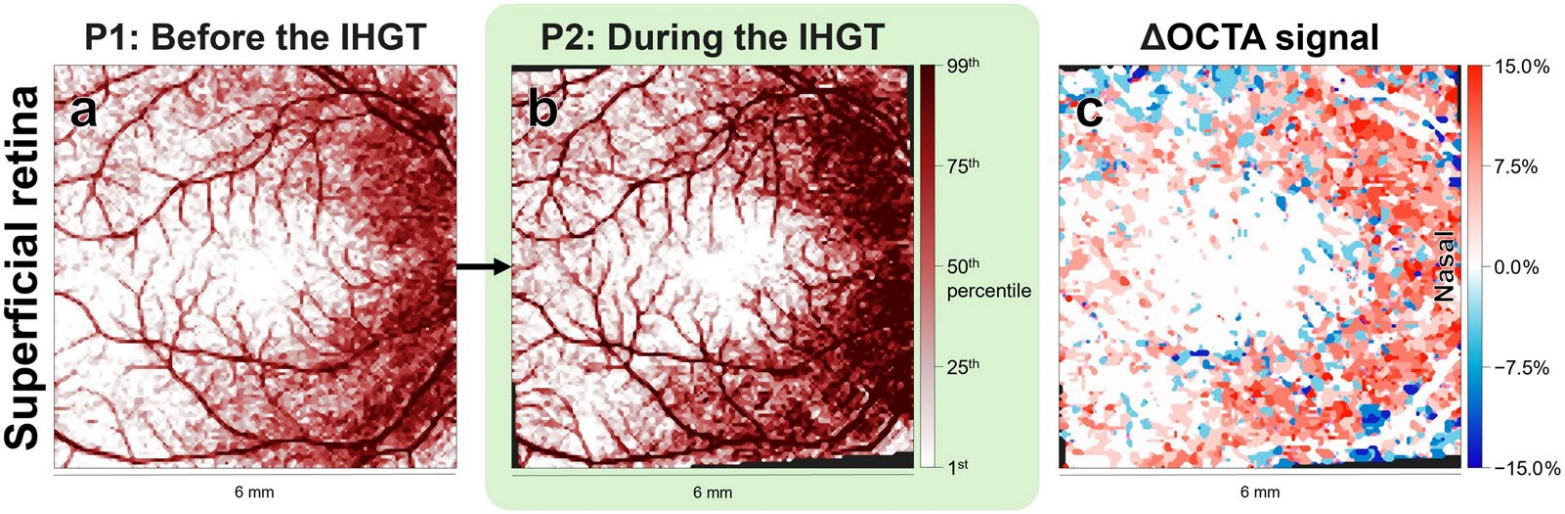
Individual example of isometric hand-grip test (IHGT)-induced change on processed optical coherence tomography angiography (OCTA) signal. Superficial retinal processed OCTA signal (%) before the IHGT (**a**) and during the IHGT (**b**), showing increased small capillary processed OCTA signal at the nasal retina. This was confirmed through topographical cluster analysis (**c**), highlighting nasal regions of increased processed OCTA signal, while sparing areas corresponding to the large vessels (white). Raw OCTA images in Figure S3

Qualitatively, there was an increase in processed OCTA signal at the nasal superficial retina (Fig. 5a, b), possibly affecting the small capillaries more so than the larger arterioles or venules. This was confirmed through topographical cluster analysis, highlighting mostly nasal regions of increased processed OCTA signal which excluded large retinal arterioles and venules (Fig. 5c).

## Discussion

This study investigated the localised effects of the IHGT on processed OCTA signal in eyes with neovascular AMD. Hand-squeezing at maximum relative grip strength for at least 30 s increased systemic BP and translated into increased processed OCTA signal at the nasal superficial retina, likely reflecting changes in the radial peripapillary capillary plexus. These changes persisted after adjusting for test–retest thresholds. Hence, the IHGT-induced OCTA response may serve as a valuable marker of vascular function, particularly in diseases affecting the inner retinal vasculature such as optic neuropathies or systemic neurovascular degenerations. These findings also warrant caution when interpreting OCTA following BP changes, such as those induced by physical activity or systemic medication changes.

### Clinical application for inducing vascular stress during OCTA

OCTA is a promising, non-invasive supplementary tool for assessing retinal-choroidal vascular integrity. This has been espoused in a recent survey of retinal specialists [34], though challenges persist in translating statistically significant but inherently ‘noisy’ research findings into clinically meaningful applications. In this regard, quantifying changes beyond test–retest thresholds [35] (defined here as the variability between baseline OCTA scans before IHGT) was essential to discern true changes in retinal-choroidal vascular health from spurious fluctuations.

Other studies have explored the potential of inducing vascular stress to augment processed OCTA signal [36, 37], demonstrating applications beyond simply improved visualisation. For example, stressors such as the IHGT or light flicker stimulation provide functional assessments of vascular reactivity [36, 37], enabling earlier detection of impairments in dynamic microvascular regulation compared to static structural measures. Notably, light flicker stimulation is being investigated for its role in measuring neuronal and vascular coupling, involving interactions among pericytes, neurons, glia, and vascular endothelial cells to regulate blood flow in response to metabolic demand. In contrast, the IHGT primarily induces a systemic vascular pressor response [37–39]. As these stressors engage distinct physiological mechanisms, combining IHGT with flicker stimulation may help distinguish deficits in autoregulation from those in neurovascular coupling, enhancing the specificity of functional vascular assessments in clinical and research settings. Comparative studies are needed to directly test these paradigms and explore their prognostic value and relevance across diseases.

Consequently, and given the sensitivity of OCTA to BP fluctuations, caution is warranted when interpreting OCTA following systemic haemodynamic changes, such as those induced by physical activity or medication changes. For instance, walking a patient to the OCTA device may transiently elevate BP, necessitating a rest period of at least 30 s to mitigate its influence on vascular measurements. Similarly, longitudinal comparisons of OCTA metrics should account for systemic BP alterations from medication changes, underlying cardiovascular conditions, or autonomic variability. Failure to account for these factors may introduce inconsistencies in quantitative OCTA interpretations, confounding disease monitoring and treatment evaluation.

### Mechanisms underlying the IHGT-induced change in vascular flow

This study reaffirmed that the IHGT induces an increase in systemic BP – a well-established sympathomimetic pressor response [10, 11]. Despite lacking autonomic innervation [40], the retina can respond to these fluctuations through myogenic and metabolic autoregulation of vascular tone [41]. This effect has been corroborated in recent reports of IHGT-induced increases in retinal blood flow in normal healthy eyes, those with mixed macular abnormalities, and eyes with central serous chorioretinopathy [8, 9, 14]. Specifically, Lupidi et al. [9] reported a 4.8% relative increase in processed OCTA signal of central serous chorioretinopathy neovascular lesions. This was comparable to the lower-end of unadjusted processed OCTA signal changes in this study (3.43%–26.42%), with the higher magnitude seen here likely attributable to the use of higher-resolution image analysis, enhancing sensitivity.

The observed correlation between OCTA signal and systolic BP change (but not diastolic) suggests that systolic peaks may dominate the retinal haemodynamic response during IHGT. Systolic BP reflects the amplitude of the cardiac pulse wave and perfusion pressure, whereas diastolic BP reflects peripheral resistance and may be more tightly autoregulated [42]. OCTA’s interscan timing also favours detection of faster flows closer to peak systolic speeds, while lower diastolic velocities may fall below sensitivity thresholds [43]. Thus, both physiological and technical factors may account for the stronger systolic association.

Topographically, IHGT-related increases localised to the nasal macula in the superficial slab, which aligns with the anatomical distribution of the radial peripapillary capillary plexus. This plexus comprises longitudinally oriented vessels within the retinal nerve fibre layer, with fewer anastomoses compared to deeper or more reticular beds, which may make it more susceptible to transient systolic perturbations such as those induced by the IHGT [44]. Its proximity to the optic nerve head and relatively short hydraulic distance to the ophthalmic artery may further enhance this susceptibility [45]. These features likely explain the preferential reactivity observed in this region.

In contrast, the deep retinal slab exhibited minimal detectable change. This may reflect both anatomical and technical factors. Anatomically, the deep capillary plexus lies farther from the radial peripapillary capillary plexus and is more so coupled to the outer retina, where neurovascular regulation is more strongly influenced by metabolic and myogenic mechanisms [44]. Technically, deeper layers in OCTA are subject to greater signal attenuation with depth and increased threshold masking, disproportionately affecting low-flow regions and reducing sensitivity to subtle changes [46, 47]. Moreover, slower perfusion rates in the deep plexus may approach the lower detection limit of OCTA, while residual projection artefacts further confound interpretation [47–49]. Although post-processing steps were applied to mitigate these limitations, they remain inherent particularly to spectral-domain OCTA. No significant signal change was observed in either the temporal macula or deep layers suggests that the IHGT may not deliver a sufficiently strong or spatially diffuse pressor stimulus to elicit widespread retinal vascular responses. This contrasts with stimuli such as flicker light, which provoke robust neurovascular coupling and have been shown to increase signal across both superficial and deep slabs [36, 37].

Additionally, the absence of significant IHGT-induced changes in the choroid was peculiar. Previous work has posited that the autonomic innervation of the choroid predisposes it to increased blood flow during isometric exercise [7, 9, 14, 50–52]. Thus, vascular alterations were expected to be more apparent in participants with systemic and ocular vascular compromise, particularly given that a higher proportion (83%) of the study population had cardiovascular-related disease [53]. This has been demonstrated in eyes with central serous chorioretinopathy via the IHGT, and neovascular AMD via the less practical squatting exercise [9, 54]. Several factors may explain the discrepancy. Use of spectral-domain rather than swept-source OCTA would have precluded wavelength penetration into the deeper choroid and hence, limited visualisation of subtle choroidal changes [55]. Furthermore, type 2 subretinal neovascularisation was more common than type 1 subretinal pigment epithelial neovascularisation in this study population. As type 1 neovascularisation is closer to the inner retina than type 2, this may have biased detection towards retinal rather than choroidal vascular changes. More work using swept-source OCTA in other populations with differing neovascular aetiologies are required to determine whether the IHGT and other isometric exercises can augment processed OCTA signal output at the choroidal vasculature, which will be useful for assessing outer retinal/choroidal diseases.

Comparative studies reinforce these interpretations. In healthy adults, isometric or dynamic exercise increased choroidal but not retinal perfusion [56], suggesting that intact retinal autoregulation limits reactivity in healthy tissue. By contrast, in older or diseased populations (including macular disease cohorts and our current study cohort), IHGT-induced induced superficial retinal changes may reflect a reduced autoregulatory reserve, age-related vascular stiffness, or both [14].

## Limitations

The primary limitation is that IHGT-induced OCTA changes were confined to the inner retina, despite studying neovascular AMD, where vascular insult typically begins in the outer retina and choroid with secondary inner retinal effects [27, 57, 58]. While this supports emerging evidence that stress-induced vascular responses may reflect the integrity of retinal autoregulation [36], it narrows the potential utility of IHGT. Instead, IHGT-augmented OCTA may be more informative in diseases with primary peripapillary or inner retinal vascular dysfunction such as glaucoma, ischaemic optic neuropathies, optic neuritis, and systemic neurovascular disorders, where impaired autoregulation is increasingly recognised [59–64].

Secondly, our within-participant design and modest sample size limited the ability to explore how individual demographics, clinical characteristics, or treatment variables influence IHGT-induced OCTA changes. For example, our prior crossover trial [14] linked reactivity to absolute grip strength, but this study was not powered to assess additional modifiers such as age, medication class, or neovascular subtype. Regressions were not performed for these covariates, as they are time-invariant and do not confound within-subject contrasts, and were further complicated by medication heterogeneity. As we did not include a concurrent healthy control group, this restricts our ability to benchmark current responses against normal physiology. Future studies with larger, phenotypically diverse cohorts (including healthy and disease-stratified groups) are needed to benchmark IHGT-induced responses across layers and support broader generalisability.

Lastly, our protocol did not include continuous OCTA acquisition (i.e., OCTA video) or comprehensive structural OCT analysis. As a result, transient vascular responses in the deeper plexuses or choroid (which may lag behind the systemic pressor response), as well as intrinsic metabolic changes in retinal neurons detectable via OCT reflectivity mapping [65–67], may have been missed.

## Conclusion

This study demonstrated that hand-squeezing at maximum grip strength temporarily increases processed OCTA signal at the nasal superficial retina, likely reflecting changes in the radial peripapillary capillary plexus. This response may serve as a valuable marker of vascular function. Consequently, caution is warranted when interpreting OCTA results following BP changes, such as those induced by physical activity or systemic medication changes.

## Supplementary Information

Additional file 1.

## Data Availability

The imaging dataset from this study is available upon reasonable request and with ethical approval.
