# Supplementary material for: Localising OCTA changes induced by the isometric hand-grip test to the superficial retina in neovascular age-related macular degeneration
Source: Eye Vis (Lond). 2025 Nov 1;12:44. doi: 10.1186/s40662-025-00459-9 (PMC12579405; doi:10.1186/s40662-025-00459-9)

Supplementary material

**Figure S1. Aggregated raw optical coherence tomography angiography (OCTA) images across periods.** Aggregated (median) raw OCTA images are shown for the superficial retina, deep retina, and choriocapillaris at **(a, c, e)** baseline period 1 (P1) before the isometric hand-grip test (IHGT), and **(b, d, f)** period 2 (P2) during the IHGT. Each panel is a median composite generated in ImageJ; no additional image processing was applied. These raw images represent the qualitative, unprocessed data for the quantitative change maps in Figures 2 and 3. Note that the superficial retina shows greater visibility nasally during the IHGT, whereas the deep retina and choriocapillaris appear qualitatively unchanged, consistent with the cluster-based analysis.


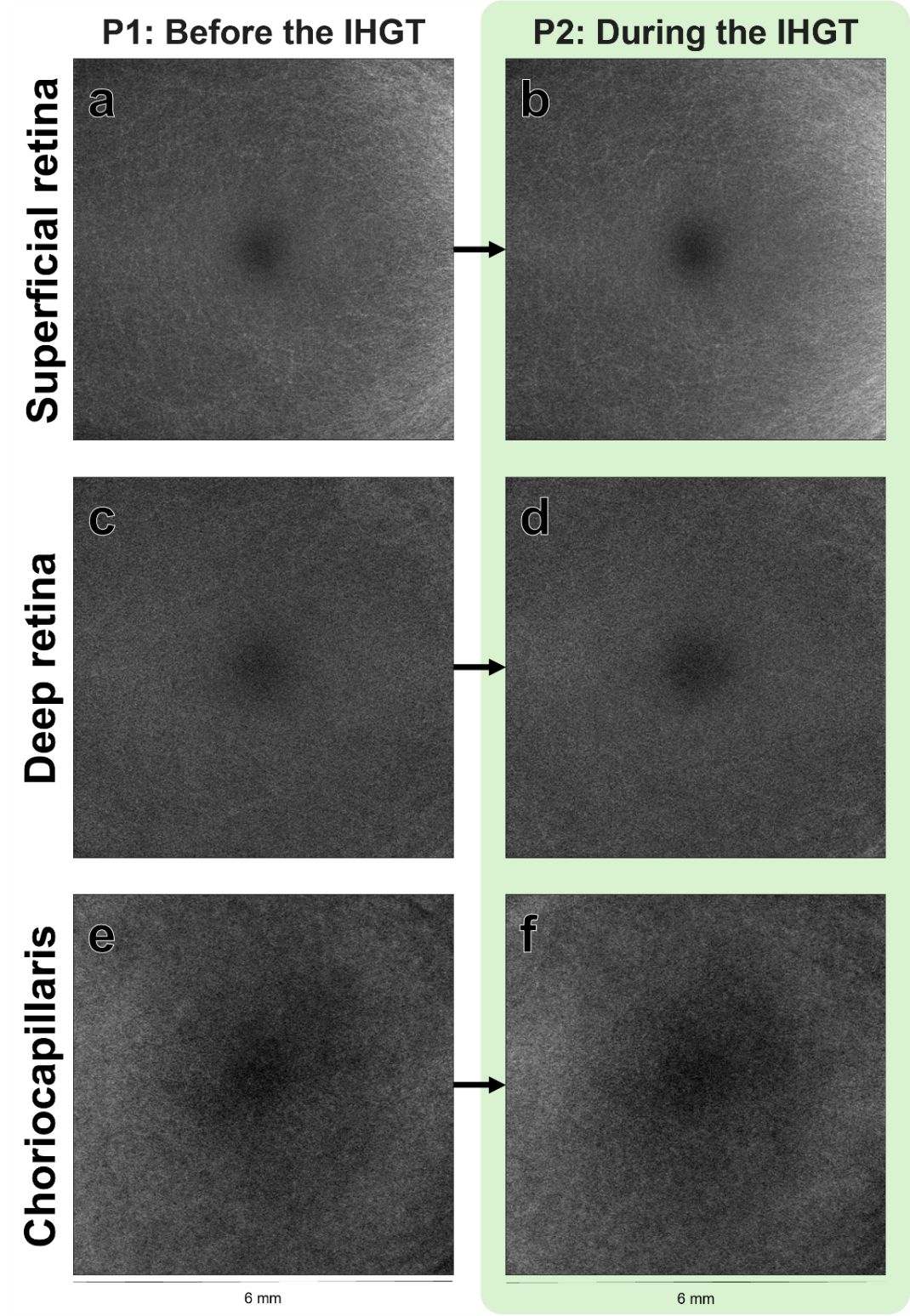


**Figure S2. Lack of change in processed optical coherence tomography angiography (OCTA) signal (%) before and after the isometric hand-grip test (IHGT), confirming adequate wash-out.** Processed OCTA signal (lack of) changes (%) are shown for the superficial retina **(a)**, deep retina **(b)**, and choriocapillaris **(c)** at baseline period 1 before the IHGT and period 3 after the IHGT. Darker red = greater increases, darker blue = greater decreases, white = no change. All values denoted as median [95% CI]. While clusters indicated some changes (*chartreuse lines*), note the presence of symmetrically distributed clusters of increased and decreased signal with similar magnitudes, centred around zero, suggesting measurement noise rather than true change. Thus, 30 seconds after IHGT was sufficient for processed OCTA signal to return to baseline.

**
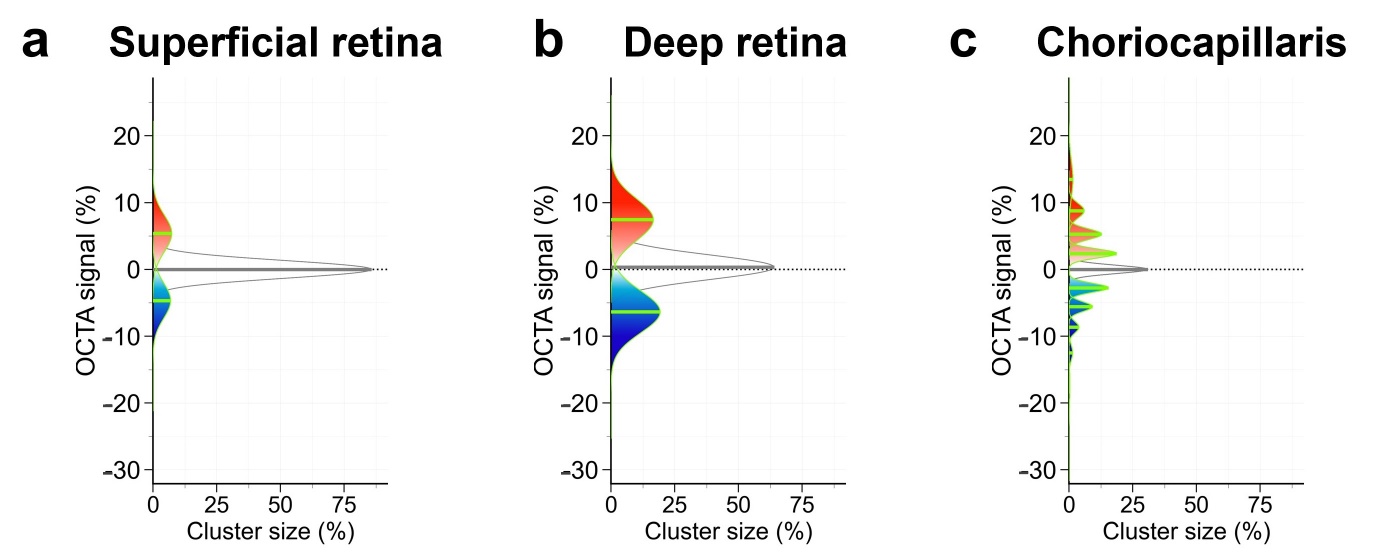
**

**Figure S3. One example of IHGT-induced change on raw optical coherence tomography angiography (OCTA) images.** Superficial retinal raw OCTA images before the isometric hand-grip test (IHGT) **(a)** and during the IHGT **(b)**, showing increased small capillary OCTA signal at the nasal retina. These raw images represent the qualitative, unprocessed data for the quantitative change maps in Figure 5. Note that the superficial retina shows greater visibility nasally during the IHGT.


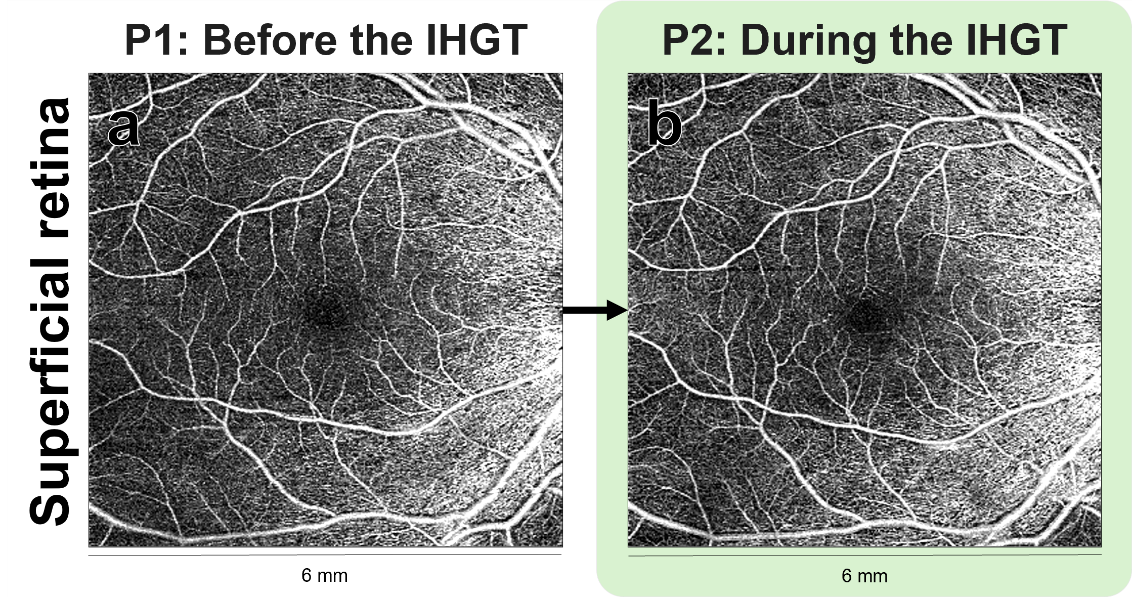

Supplement: Supplementary file 1 — Additional file 1. [file 40662_2025_459_MOESM1_ESM.docx]
